# The development of the People with Aphasia and Other Layperson Involvement (PAOLI) framework for guiding patient and public involvement (PPI) in aphasia research

**DOI:** 10.1186/s40900-023-00484-9

**Published:** 2023-09-01

**Authors:** Marina Charalambous, Alexia Kountouri, Jürg Rainer Schwyter, Jean-Marie Annoni, Maria Kambanaros

**Affiliations:** 1 Laboratory of Cognitive and Neurological Sciences, Department of Neurology, University of Fribourg, Chemin du Musée 8, 1700 Fribourg, Switzerland; 2 Present Address: The Brain and Neurorehabilitation Lab, Department of Rehabilitation Sciences, Cyprus University of Technology, 30 Arch. Kyprianos Str, 3036 Limassol, Cyprus; 3 Stoke Ambassador, Cyprus Stroke Association, Limassol, Cyprus; 4 Formerly Professor of English Linguistics, University of Lausanne, Lausanne, Switzerland; 5 Allied Health and Human Performance, University of South Australia, Adelaide, SA Australia

**Keywords:** People with aphasia, Patient and public involvement, Framework, Co-design, Stroke

## Abstract

**Background:**

Patient and Public Involvement (PPI) in aphasia research requires researchers to include people with aphasia as research partners from the beginning of the study. Yet the quality of reporting on the level and type of involvement is poorly documented in the absence of a framework to guide PPI in aphasia research. This study aimed to extract the items and statements relevant for the development of the People with Aphasia and Other Layperson Involvement (PAOLI) framework for designing and implementing PPI in aphasia research, in collaboration with people with aphasia.

**Method:**

The method recommended by the EQUATOR network was followed. This involved: (1) evidence from a scoping review, (2) a thematic analysis of the in-depth interviews, of people with stroke and aphasia, on the topics to be included in the pilot draft, (3) a two round Delphi survey for item/statement selection and (4) an experts’ consensus meeting. The research team involved two PPI partners with chronic stroke-induced aphasia. The research process involved co-design and was informed by the Dialogue model.

**Results:**

Twenty-three panellists, from 13 countries, voted in round one with 87% (20/23) responding in round two. The final PAOLI framework includes the following 17 items (with 66 descriptive statements): establish collaborations, recruit patients, gain informed consent, organize induction meetings, train patient partners, create communication links, engage communication partners, conceptualize topics, establish research priorities, reach consensus, work with co-design methods, develop proposals, assist with dissemination of results, promote implementation of the outcomes, support patient partners and promote self-evaluation, monitor progress and assess impact of the patient involvement. These items were considered by the panellists as the most relevant for the involvement of people with aphasia as research partners.

**Conclusion:**

The PAOLI is the first international consensus framework for guiding patient involvement in aphasia research. Researchers are encouraged to adopt the framework to improve the quality of their research by promoting the meaningful involvement of people with aphasia within the research team from the start.

## Abstract (plain language summary)

Aphasia is a communication disorder which results in challenges with everyday interactions and impacts quality of life. Qualitative research involving people with aphasia often investigates quality of life. Until very recently people with aphasia were either excluded from such research teams or occasionally included as consultants but without their contribution documented or reported by the research team. The current work builds on evidence that has identified the absence of a standardized approach for designing and implementing the active involvement of people with aphasia in aphasia research teams. This approach was informed by patient participation involvement principles and the Dialogue model that involves engaging with patients/clients about such issues. This prompted the creation of the People with Aphasia and Other Layperson Involvement (PAOLI) framework, in close collaboration with people with aphasia. The research team involved two patient partners with chronic aphasia after stroke.

To decide on the content to be included in the PAOLI framework a two-round international voting (Delphi survey), with 23 panellists from 13 different countries, and a meeting to finalize the framework were completed. The PAOLI includes 17 items (with 66 descriptive statements) on how to: establish collaborations, recruit patients, gain informed consent, organize induction meetings, train patient partners, create communication links, engage communication partners, conceptualize topics, establish research priorities, reach consensus, work with co-design methods, develop proposals, assist with dissemination of results, promote implementation of the outcomes, support patient partners and promote self-evaluation, monitor progress and assess impact of the patient involvement. These items were considered by the panellists as the most important for the involvement of people with aphasia as partners in research teams.

The PAOLI is the first international framework for guiding patient involvement in aphasia research. Researchers are encouraged to use the PAOLI framework to improve the quality of their research by supporting the meaningful involvement of people with aphasia within their research team.

## Introduction

Aphasia is an acquired communication disorder caused by damage to the language areas of the brain mainly resulting from a stroke [1]. Approximately 21–40% of stroke patients sustain permanent aphasia, that becomes chronic with time, having a significant impact on quality of life and rehabilitation outcomes [2]. Out of 60 major diseases and 15 health conditions, aphasia was identified as having the worst effect on quality of life, even when compared with cancer and dementia [3]. For this reason, the impact of aphasia on the lives of people living with chronic stroke, has been studied extensively by qualitative research teams [4].

Nevertheless, until very recently people with aphasia (PWA) and their significant others (the public) were excluded as research partners from teams carrying out the research [5, 6]; especially when the communication impairment was not the primary goal of the project [7]. Consequently, the relevance and the impact of the research was compromised [8]. Over time aphasiologists have suggested various strategies and techniques to promote the inclusion of PWA in research teams [8]. Indeed, their inclusion in stroke rehabilitation studies has increased as evidenced in allied health research: physiotherapy [9], occupational therapy [10, 11], psychology [12] and multidisciplinary research [13]. Unfortunately, research so far involving PWA as research partners has revealed their involvement as tokenistic and nonmeaningful [8]. For this reason, research funding bodies mandate the involvement of PWA and the public not only as (passive) research participants, but also as research partners coined as patient and public involvement (PPI) [14].

Patient and Public Involvement (PPI) is the required need for the direct and active involvement of PWA (and the public) within the research team, from the onset of the research process. This approach ensures the democratic representation of PWA in research teams, that the research is ethically responsible and relevant, and that wasting valuable resources is minimized [15]. Funding agencies and ethical review boards also endorse the inclusion of national aphasia associations, support organizations, advocates, and policymakers in the research submissions to ensure the sustainability and broad dissemination of the research results [16].

Patient and public involvement involves various participatory approaches and includes a range of different activities throughout the research procedure [8]. In 1969, Sherry Arnstein published an influential paper titled ‘A Ladder of Citizen Participation’. In this paper, Arnstein [17] described a hierarchy of participation, figuratively as a ladder, starting at the lowest rung (level) of non-participation, such as manipulation through tokenism, up to various degrees of citizen control. At the top of the ladder, patient partners are involved in the research team with autonomy and democratic structures [17].

As there is no ‘one size fits all’ approach to conducting PPI research with PWA we initiated this project by conducting a review to explore whether PWA were involved as research partners in the creation of patient reported outcome measures (PROMS) exploring quality of life with aphasia. These PROMs are questionnaires that collect data to report and monitor patients’ subjective assessments of their symptoms, functional status, and quality of life [18]. Twenty published studies, from the review, showed significant limitations around the reporting of the design, context, and the process of PPI, deterring the interpretation of the PPI impact [14]. Specifically, the results revealed that during the creation of these tools PWA were either excluded from research teams or were placed on one of the lowest rungs of the PPI ladder (tokenistic approach). Overall, it appears that there is a mismatch between the items chosen by researchers in the tools, and the pragmatic needs of PWA. We proposed that the role of PWA, in the published studies, was mostly consultative in nature in the absence of a framework to guide the involvement of PWA. The absence of a standardized approach for designing and implementing PPI in aphasia research prompted the authors in 2020 to develop a framework specific to involving people with aphasia in research, that is, “the People with Aphasia and Other Layperson Involvement” (PAOLI) framework.

The EQUATOR (Enhancing the QUAlity and Transparency Of health Research) network [19] has developed high standards in PPI reporting with the Guidance for Reporting Involvement of Patients and Public (GRIPP2) checklist by Staniszewska and colleagues [20]. The GRIPP2 aims for researchers to design all-purpose PPI methods, and to be used as a quality assurance measure in the documentation of PPI in the scientific publication. The recent review by Jones et al. [21] on exploring the reporting of PPI using the GRIPP2, by the co-researchers of the team (4 patient partners living with chronic conditions), reported the GRIPP2 to be complicated and not user-friendly [21]. Furthermore, the critique of human rights-based approaches to health research embraces that generic PPI frameworks, might act as tokenistic and not be “accessible” or applicable to all patient populations [22] especially those with persisting communication difficulties like PWA [14].

It was important for this project that the insights of PWA are “voiced” to help the team identify important dimensions that may be overlooked by researchers, leading to more comprehensive and valid outcomes. The involvement of PWA during the codesign stage can provide valuable input on the acceptability and feasibility of the research questions. PWA can assess whether the language, wording, or response options of the materials are clear, easy to understand, and are culturally appropriate. The input from PWA can help identify potential barriers to research completion, or limitations in certain areas of the research process, enabling researchers to refine and improve their outcomes to maximize the usability and acceptability of the research outcome/end-product. Therefore, aphasia, and the corresponding communication difficulties, make it ideal to develop a methodological PPI framework like the PAOLI.

Before initiating the development of the PAOLI, we published a thematic analysis on the exploration of the views of people living with chronic stroke and aphasia on their potential involvement as research partners [23]. For this project we interviewed the patient partners of this study, who presented with experience in research prior to the stroke event. Given the innovative phase of this process, we expected that their understanding of the research process in combination with their lived stroke/aphasia experience, would assist them to anticipate possible barriers and facilitators that they might face when involved in the PAOLI study. The research team included a patient partner with stroke-induced chronic aphasia and her communication partner. The results of the thematic analysis revealed four areas that may impact the participation of PWA in research teams and include: (1) the *Restrictions* that make their involvement challenging, (2) the levels and manner of *Involvement*, (3) the *Support* required for meaningful contribution, and (4) the *Impact* of their input on the research outcome. These four areas served as the key topics during the PAOLI codesign phase [23]. The BEFORE recommendations also developed from the thematic analysis research, and reported below, were considered before commencing the PPI study with PWA as research partners [23]. The BEFORE recommendations are as follows:

1. Build rapport by having regular one-on-one meetings with PWA and other stakeholders to explain research commitments and offer information on the project.
2. Establish the communication needs of PWA and create appropriate communication ramps before their involvement e.g., have short meetings and create accessible materials.
3. Foster a robust support system by recruiting communication partners that can assist PWA throughout the research process or when needed.
4. Offer accessible online training sessions upon request in relation to the PPI project (e.g., what is a Delphi study and how they would be involved).
5. Reinforce the use of tailored resources such as simplified material and resources designed in collaboration with PWA
6. Encourage patient partners with aphasia to participate in aphasia support groups to improve communication and social skills to foster more active engagement within the research team.

PAOLI strives to help researchers support the active involvement in the research team, of people with communication impairment. It incorporates the principles of PPI while formulating a “patient-partner centred” approach of PPI design and implementation [24]. Importantly, this framework aims to counterbalance the power dynamics within the research team and encourage researchers to respond to the personalized needs of the patient partners [25]. However, this does not imply that the patient partners are passive in the process, but rather active stakeholders aiming for greater equality and collaboration in the research process. This will enable PWA to have ‘a research voice’, to express their views, participate actively in decision-making, and be represented from early on, ultimately improving the impact of the research given that PWA are most often the end-users of the research outcomes/product [8].

## Methods

For the development of the PAOLI, the EQUATOR (Enhancing the QUAlity and Transparency Of health Research) network’s recommended method for developing a methodological framework [24] was followed. This involved rapid evidence synthesis (gathered evidence from a scoping review) [14] and a Delphi consensus practice [24]. This included a two-round Delphi survey for item/statement selection and an experts’ consensus meeting to finalize the framework. As mentioned above, prior to the Delphi study, we conducted interviews with PWA and stroke and had undertaken a thematic analysis to identify topics to be included in the framework [23]. PAOLI was registered on the EQUATOR Network website in June 2022 (https://www.equator-network.org/library/reporting-guidelines-under-development [accessed 10 December 2022]).

**Fig. 1:**
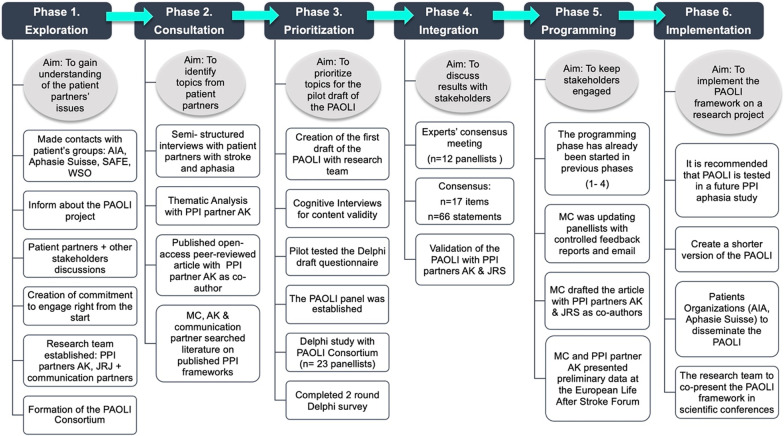
Overview of the six phases of the dialogue model for the development of the PAOLI. Note: AIA = Association Internationale Aphasie, SAFE = Stroke Alliance for Europe, WSO = World Stroke Organization

To begin the PAOLI development process, the *Dialogue Model* [26], a multi‐phased PPI scheme, based on the methodology of the Responsive Evaluation [27] and the Interactive Learning and Action approach [28], was selected. The rationale for selecting the Dialogue Model for research agenda-setting, was that it enables an equal partnership to be created between all stakeholders (including patients) during the research process. The first author (MC), the lead investigator, served as the facilitator. The Dialogue Model has six phases: Exploration, Consultation, Prioritization, Integration, Programming, and Implementation. These phases are presented in Fig. 1 with a description of the actions related to the development of the PAOLI framework.

### Dialogue model phase 1. Exploration

The *Exploration* phase involved contacting patient organizations, such as the Association Internationale Aphasie, the Stroke Alliance for Europe, the World Stroke Organization, to inform potential stakeholders about the PAOLI project, recruit panellists, form the PAOLI consortium and establish the research team. Specifically, prior to commencing the PAOLI study, and to accommodate the needs of PWA for active involvement and promote their autonomy [29], we applied the BEFORE recommendations, resulting from the recent thematic analysis [23]. This included one- on-one meetings with PWA and other stakeholders to explain the PAOLI project. To create the necessary infrastructure to support PWA, communication partners were recruited and simplified information was provided before the initiation of the study. All materials (consent forms, invitation letters etc.) were prepared in accessible formats, in collaboration with co-author AK (see Appendix 1). Two patient partners requested personal meetings to receive further information on the Delphi method and what they needed to do. Most but not all patient partners with aphasia were members of aphasia communication groups in their respective countries. The others who were not, were prompted to join their respective groups, so that the experience of communication group membership, would boost more active engagement with the other research team members [30].

### The research team

The research team consisted of 7 individuals: the leading investigator, 2 PPI partners and 4 communication partners. The first author (MC), a senior speech and language therapist practising in aphasia rehabilitation, with previous PPI experience in aphasia research^1^ served as the lead investigator [14, 23, 31]. The two PPI partners are co-authors AK and JRS. The term ‘PPI partner’ will be used to reflect the constant commitment and active involvement of the two people with chronic stroke-induced aphasia throughout the research process. The aim for collaborating with AK and JRS was to enhance the quality and transparency of the PPI evidence in this study, by involving them from the beginning and in all stages of developing PAOLI. AK is a 36-year-old female with mild-moderate aphasia and a right hemiparesis, following a hemorrhagic stroke 8 years prior. AK holds a Master of Science (MSc) in Social Research Methods from the University of Sussex and was enrolled in doctoral studies in Social Care but dropped out after her stroke event. She has previous experience as a PPI partner on research projects and has co-authored publications from the research [23, 31]. JRS was Professor of English Linguistics at the University of Lausanne and is now retired. He suffered a stroke in 2009 and presents with mild-moderate aphasia and a right hemiplegia. JRS holds a PhD in Linguistics from the University of Cambridge, has a vast background in research as primary investigator, on several projects, and has authored aphasia-related research publications [32–34]. Both AK and JRS were actively involved in all stages of this study, from the conceptualization of the study to patient recruitment and to the final validation of PAOLI. Finally, the research team included four laypeople the so-called communication partners (family members, speech-language therapy (SLT) students) who supported PWA from the onset during the informed consent procedure, the completion of the cognitive interviews, the Delphi survey, and the validation phase.

### Dialogue model phase 2. Consultation

The *Consultation* phase involved 8 semi-structured interviews with people with chronic stroke, four with aphasia and four without. These interviews were subject to a thematic analysis from which the key topics to be included in the pilot draft of the PAOLI were identified. [23] The Consultation phase involved the publication of the results of the thematic analysis, in an open-access peer-reviewed journal, with the PPI partner (AK) as co-researcher and co-author. Also, a literature search on published PPI frameworks [22] was completed by the research team (RT)**.** The RT consulted several published guidelines and frameworks [22, 35] to become familiar with recommendations from previous research on the conceptual elements required to build a new framework. Specifically, the RT accessed the following:

- the patient and service user engagement (PSUE) framework [36] on how to develop the research phases.
- the European Alliance of Associations for Rheumatology (EULAR) recommendations [37] to determine topics on conceptualization, researching consensus and co-design methodology.
- the research processes from the Enhancing the Quality of User Involvement in care Planning (EQUIP) study [38, 39] to gather information on patient recruitment and induction procedures.
- the Guidance for Reporting Involvement of Patients and the Public (GRIPP2) checklist [20] on selected material regarding proposal development and impact.

### Dialogue model phase 3. Prioritization

All this information combined, resulted in the conceptual development of the PAOLI leading to the adoption of 18 items and 99 statements and the creation of the first pilot draft. For this study items are defined as the minimum set of information that compose and define a set of statements [40]; whereas statements are the detailed components of the item. We did not insist on formulating “aphasia-friendly materials” in line with the published studies [41, 42] but mostly focused on co-writing and co-designing with PWA comprehensible statements and written material to promote their reading comprehension and engagement in the study [43]. The pilot draft of the PAOLI encompassed the following components:

- Four phases: Foundation, Development, Translational and Ongoing Processes.
- 18 items [9] which included the following 99 statements:
  - 33 statements in *Phase I: Foundation*, on establishing collaborations, recruiting patients, gaining informed consent, organizing induction meetings, training patient partners, creating communication links and engaging communication partners.
  - 34 statements in *Phase II: Development*, on conceptualizing topics, establishing research priorities, reaching consensus, working with co-design methods, and developing research proposals.
  - 18 statements in *Phase III: Translational*, on data analysis, assisting with the dissemination of the results and promoting the implementation of the outcomes.
  - 14 statements in *Phase IV: Ongoing Processes*, on supporting patient partners and promoting self-evaluation, monitoring progress, and assessing the impact of the patient involvement.

After organizing the conceptual constructs, the research team formulated the 99 statements using a 5-point Likert Scale by asking, “How important is it to…” (1 = not important to 4 = very important 5 = extremely important) using an accessible format selected by AK as appropriate for PWA.

### Cognitive interviews and pilot test

After the first draft was created, two rounds of cognitive interviewing and a pilot test to explore how patient partners with aphasia understood the statements were completed. The cognitive interview study aimed to improve the validity and acceptability of the questionnaire and followed the Consensus-based Standards for the selection of health Measurement Instruments (COSMIN) methodology [44]. This method was followed to ensure a structured process while testing the content’s relevance. Participants for this part of the study were PWA outside of the research team who were involved only in the testing stage of the pilot draft. For the completion of the cognitive interviews PWA were recruited from the aphasia communication group at the University Rehabilitation Clinic of the Cyprus University of Technology. The interviews were completed by author MC with the occasional assistance of the communication partners (final year SLT students). Signed informed consent was received from PWA prior to the initiation of the interviews. The first cognitive interview was performed with a retired person with moderate anomic stroke-induced aphasia (SO). During the first cognitive interview SO completed the questionnaire under the guidance of author MC. On completion, SO proposed modifications to 11 statements in terms of simplifying the vocabulary (e.g., replace ‘tailor made’ with ‘personalized’) and the complexity of the statements (e.g., replace ‘PWA to form a steering committee to check, and feedback, on the progress of the research’ with ‘PWA to form a committee to check the progress of the research’). Once modifications were made to the first draft of the questionnaire, the second cognitive interview was undertaken with MG. MG who had suffered a left hemispheric stroke and presented with mild-moderate anomic aphasia. MG approved the revised content, and proposed an additional statement as follows: ‘PWA to designate as a communication partner another person with aphasia’ as she felt that PWA can support each other very effectively during group discussions. This resulted into 100 statements for the Delphi survey. The questionnaire with the 18 items and the 100 statements is reported in Appendix 2.

Since the first two pilot tests were face-to-face, a third pilot was completed to test the method online. During the final pilot, DT a person presenting with stroke-induced expressive aphasia completed the questionnaire online. DT recommended that instructions in the email be simplified regarding accessing, completing, and submitting the questionnaire. These issues were addressed before the initiation of the Delphi study. See screenshot in Appendix 3 of the accessible online version of the Delphi survey.

### Delphi study

The Delphi technique is widely used to achieve reliable consensus from a group of subject experts on a particular issue [45]. For this study, the Delphi technique:

- Supported the anonymity of the panellists and the confidentiality of their responses;
- Brought together a geographically dispersed international panel of experts;
- Encouraged honest opinion;
- Allowed a structured/organized group communication process;
- Facilitated endorsement of the computer-based survey by the PPI partners (AK and JRS) as simple to use;
- Was a method free from group pressure, especially for patient partners [27].

The “Recommendations for the Conducting and Reporting of Delphi Studies” (CREDES) [45] was followed (see Appendix 4 for the CREDES checklist). Based on the judgment and discretion of the authors of this study, to ensure a wide scope of opinion related to developing the PAOLI, the expert panel was categorized as follows:

- PPI and aphasia experts in academia, scientific publications, and training;
- “Experts by experience” who were PWA and stroke survivors without aphasia (SswoA: we aimed for the opinion of people with stroke and unaffected language skills but were experiencing other symptoms of stroke i.e., fatigue, hemiplegia etc.);
- Aphasia rehabilitation clinicians;
- Stroke and aphasia advocates;
- Stroke policy makers;
- The author of previous PPI guidelines/frameworks/recommendations; and a
- Research funding agent.

The authors (MC, JMA and MK) identified experts in the field of PPI, stroke and aphasia research and rehabilitation through a nomination process. People with stroke and aphasia were also nominated and recruited by MC, AK and JRS in Europe through a snowball effect. Individuals were considered eligible to be invited to participate in the expert panel if they had relevant clinical and or/academic backgrounds and experiences concerning PPI in stroke and aphasia research and/or rehabilitation and could contribute to the topic of the study.

The criteria for the patient partners were as follows: (1) to be a stroke survivor, (2) to be in the chronic stage of stroke (> 6 months post-stroke), (3) to be able to speak, understand, read, and write in English post-stroke, (4) to be socially active as confirmed from case history, (5) to have at least one academic qualification (Bachelor), and (6) to have had previous research experience, either as a student or as a researcher. Furthermore, for PWA evidence from case history interview of mild-moderate chronic aphasia was a criterion. The Aphasia Severity Rating Scale (ASRS), of the Boston Diagnostic Aphasia Examination (BDAE) [46] was used to rate the severity of the observed language difficulties. Spontaneous speech samples were elicited during a 15-min semi-structured interview that comprised of four topics: the illness, previous/current occupation, family and housing, hobbies [46]. Aphasia severity was assessed by a professional SLT, author MC, using the ASRS to allow a classification based on fluency and intelligibility. During the “nomination” process several people with aphasia rejected the invitation as they considered the topic as “too specific” or that they did not have “adequate experience” on research to understand and contribute to our project.

### Panellists

The panel consisted of 23 panellists deemed sufficient for the survey [47]. Geographical diversity was achieved by recruiting panellists from 13 different countries and various organizations around the world. Demographic characteristics of the panellists are reported in Table 1.

**Table 1:** The demographic characteristics of panellists in the Delphi study

Characteristics of the panelists	Number of panelists	
	Round one N = 23 (%)	Round two N = 20 (%)
*Gender*		
Female	16 (70)	13 (65)
Male	7 (30)	7 (25)
*Country*		
Argentina	1 (5)	−
Australia	2 (8)	2 (10)
Cyprus	5 (20)	5 (25)
Denmark	1 (5)	1 (5)
Estonia	1 (5)	1 (5)
France	1 (5)	1 (5)
Germany	1 (5)	1 (5)
Greece	1 (5)	–
Ireland	1 (5)	1 (5)
Norway	1 (5)	1 (5)
Portugal	2 (8)	2 (10)
Switzerland	2 (8)	1 (5)
United Kingdom	4 (16)	4 (20)
Roles and self-reported job titles*		
People with aphasia after stroke	5 (24)	4 (20)
Stroke survivors no aphasia	4 (18)	3 (15)
PPI Aphasia Experts	3 (12)	3 (15)
Academic Aphasia Researchers	4 (18)	3 (15)
Aphasia Rehabilitation Clinician	1 (4)	1 (5)
Clinical Psychologist on PPI	1 (4)	1 (5)
Stroke Policy Maker	1 (4)	1 (5)
Aphasia Advocate	1 (4)	1 (5)
Stroke Advocate	1 (4)	1 (5)
PPI Guidelines Author/ Policy Maker	1 (4)	1 (5)
Research Funder	1 (4)	1 (5)
*Years of experience in research*		
Less than 10	6 (25)	5 (25)
10 to 25 years	8 (35)	7 (35)
More than 25 years	9 (40)	8 (40)
*Years of experience in PPI research methods*		
None	12 (50)	9 (45)
Less than 10	2 (10)	2 (10)
10–25 years	9 (40)	9 (45)

*Note that some panellists held multiple roles in addition to their principal job title

Patient partners were enlisted from the Cyprus Stroke Association, the French Association S’ Adapter- AVC et Aphasie, the Portugal AVC Stroke Association, the Norwegian Stroke Association, Aphasie Suisse and the Stroke Association UK. Nine people with chronic stroke, five with chronic aphasia and four without, met the inclusion criteria. Patient partners were aged between 27 and 70 years old, with a range of education of 15–22 years. All patient partners had completed a research project during their studies or work commitments prior to the stroke. Specifically, PWA 3 was the primary investigator in several projects throughout his academic career and two SSwoA (1 and 4) are now the primary investigators in studies in their perspective fields. The remaining patient partners were familiar with the research process because of prior experience from the completion of thesis work while studying. The demographic characteristics of the PWA and the SSwoA are reported in Table 2.

**Table 2:** Patient partner demographics

	Gender	Stroke type (Hemiplegia)	ASRS* (0–5)	Completed education	Research experience	Premorbid empl/ment (RtW*)
*People with aphasia (PWA)*						
PWA1	Female	Haemorrhagic LH* (Yes)	4	Doctoral	Thesis Completion	Teacher (No)
PWA2	Male	Ischemic LH* (Yes)	4	Masters	Thesis Completion	Lawyer (No)
PWA3	Male	Ischemic LH* (Yes)	5	Doctoral	Primary Investigator	Academic (No)
PWA4	Female	Ischemic LH* (No)	5	Masters	Thesis Completion	Admin (No)
PWA5	Male	Ischemic LH* (Yes)	4	Bachelor	Thesis Completion	Businessman (Retired)
*Stroke Survivors without Aphasia (SSwoA)*						
SSwo1	Male	Ischemic LH* (Yes)	N/A	Doctoral	Primary Investigator	Academic (Yes)
SSwo 2	Female	Ischemic LH* (No)	N/A	Masters	Thesis Completion	Nurse (Yes)
SSwo3	Female	Ischemic LH* (No)	N/A	Bachelor	Thesis Completion	Unemployed (No)
SSwo4	Female	Ischemic LH* (No)	N/A	Doctoral	Primary Investigator	Academic (Yes)

*LH left hemisphere, *ASRS* aphasia severity rating scale: 0 = limited verbal output and comprehension, 5 = mild word finding difficulties, *RTW* return to work

### Delphi survey

A consensus level of 80% was selected as this percentage marks a clear majority opinion [48] and was used in previous PPI framework development research using the Delphi method [20]. Therefore, a statement was deemed to be ‘very important’ or ‘extremely important’ (it was considered as the most useful for the involvement of PWA as research partners for the PAOLI framework) if it had been rated as either 4 or 5 on the Likert scale by at least 80% of respondents. In recognition that the 80% cut-off criteria selected is a strict and somewhat arbitrary definition of consensus, the statements that at least 70% of the panellists scored 4 or 5 on the Likert scale were highlighted in the findings as statements that were ‘nearing’ the pre-set cut off point and were voted on again in round two. Statements ‘nearing’ consensus from round one, that did not receive at or more than 80% consensus in round two, were eventually discarded.

In round one, panellists received an electronic invitation, with an attached link and simplified instructions. While rating all statements in each section, panellists were also asked to comment on each statement, if they wished, in the space provided. Each panellist was allowed 4 weeks to respond to the survey questionnaire [49]. After receiving panellists’ responses, the new information collected was used to modify the second version of the questionnaire. To avoid directly or indirectly influencing the experts’ judgements, MC maintained the anonymity and confidentiality of the panellists by communicating solely with each individual panel member via email. The panellists received a personalised report showing quantitative responses to the round one statements (controlled feedback report). MC examined the anonymised quantitative scores and qualitative comments for each statement and generated round two.

In round two each panellist was asked to review the statements summarized based on the information provided in round one and to rate them again using the Likert scale. As a result of round two, there was a high level of agreement for most statements and consensus was achieved. Again, after round two, MC send out a summary report to the panel with the revised set of statements.

### Data analysis

The statistical measures used for analysis were measures of central tendency (medians and mode), widely applied in Delphi studies [49] for the collective judgments of the respondents [47]. All statistical analyses were undertaken with the jamovi (version 1.6) statistics computer software [50, 51]. To prevent bias, an independent researcher was employed to statistically analyse the results of the Delphi survey.

## Results

### Results of the Delphi survey

During round one, 23/23 (100%) of the panellists responded to the initial questionnaire with statements (*n* = 56) reaching consensus > 80%, 40 statements ‘nearing’ consensus > 70% and 4 statements discarded < 69%. During round two, 20/23 (85%) of the panellists re-voted on the 40 statements that neared consensus from round one, with 10 statements meeting the consensus criterion (> 80%). From the 18 initial items only 1 item, ‘Data analysis’, was discarded as it did not receive adequate votes from the panelists. The quality of this Delphi survey was increased by the quick turnaround (4 weeks) between the two rounds [44]. This enabled swift agreement from the diverse group of panellists with commitment to the project and excellent response rates (100% for round one and 85% for round two). See Appendix 5 for the voting results of each round.

### Statements with the highest consensus level

Consensus levels were the highest in relation to the need to document the involvement of PWA in the research process from the preparation phase to the conceptualization of the topic, and the dissemination of the outcomes. Specifically, these included establishing collaborations with aphasia organizations and practice-based research networks, preparing research materials and resources using aphasia-friendly formats, and providing training sessions to patient partners on PPI design and processes. Also, co-design methodologies and deciding on a specific topic that is mutually important and interesting to explore, both for researchers and patient partners, were also voted with very high consensus. Panelists further considered as extremely important the creation of ethical and responsible research and the clear acknowledgment of the work delivered by/from patients by referring to them as patient partners when writing the research proposals (avoiding tokenism). See Table 3 for the statements with the highest consensus level**.**

**Table 3:** Statements with the highest consensus level from both rounds

Items	Statement description How important is it to:	Round one scores		Round two scores	
		Median	% of total	Median	% of total
Establishing collaborations	Make contact with local and national aphasia organisations	5.00	95	5.00	95
	Establish collaborations with practice-based research networks and community clinicians	5.00	90	5.00	90
Recruitment	Develop an accessible information leaflet in collaboration with PWA	5.00	95	5.00	95
	Create accessible invitation letters in collaboration with PWA	5.00	90	5.00	90
Gaining informed consent	Prepare accessible consent forms in collaboration with PWA	5.00	90	5.00	80
Patient partner training	Prepare and present accessible training sessions about the research design and process	5.00	90	5.00	95
	Confirm that the training is accessible to the communication needs of PWA	4.00	90	4.00	95
Conceptualisation	Generate ideas from conversations and in- depth interviews	5.00	90	5.00	90
	Run focus groups with PWA as facilitators to identify topics	4.00	80	4.00	90
Establishing research priorities	Promote patient centeredness and a focus on specific concerns	5.00	90	5.00	95
	Examine the areas of concern as revealed by PWA	5.00	90	5.00	85
Reaching consensus	Identify the topics most important to PWA	5.00	100	5.00	100
	Set the research question(s) with PWA in a manner comprehensible to all partners	4.00	95	4.00	95
	Make the aim of the study easy to understand for all partners	5.00	100	5.00	100
Proposal development	Comply with standards for ethically responsible research	5.00	90	5.00	90
	List PWA as named research partners	5.00	90	5.00	90
Outcomes and implementation	Present case studies of PWA experiences to suggest potential areas of improvements in research methodology	4.00	90	4.00	90
Dissemination and sustainability	Involve national aphasia associations and stroke support groups in the dissemination of the results	5.00	90	5.00	95

### Dialogue model phase 4. Integration

#### Experts’ meeting

During the *Integration* phase the aim was to discuss the results of the Delphi survey with the panellists in an expert’s consensus meeting [49]. This was then followed by the validation phase of the framework initiated by PPI partners AK and JRS.

The experts meeting (n = 12 panellists) involved the co-author PPI partners AK and JRS, a patient partner with stroke and no aphasia, three aphasia academic experts, an aphasia researcher, an aphasia rehabilitation clinician, a stroke advocate from the World Stroke Organization, a policy maker from Stroke Alliance for Europe, the author of the EULAR PPI recommendations and MC serving as the facilitator of the meeting. Before the experts meeting, each panellist received a report that included all statements and ratings from the two rounds. The meeting was held via the Zoom-online conferencing platform, for 90 min and gave the panellists the opportunity to further discuss the importance of the statements and the structure of the framework. MC presented the trajectory of the development of the PAOLI framework with the results from each round. The experts discussed the topics and statements that did not reach consensus, e.g., the fact that no statement from the section ‘*Data analysis and Interpretation’* was voted on in any of the two rounds. The group discussed this issue at length and agreed that *data analysis* should not be included in the PAOLI framework as it was considered a complex task for PWA. Also, AK and JRS and the patient partner with stroke, expressed their views on PPI and gave feedback on their experience of this procedure. The PPI academic experts and patient advocates shared their views on the challenges of PPI in aphasia research. The *n* = 66 statements were confirmed within the experts’ consensus meeting. See the experts’ meeting outcomes in Appendix 6.

For this Delphi study two iterations and one experts’ meeting (March 2020- Dec 2020) were sufficient to collect the information needed to reach consensus [49]. See the flowchart for the Delphi procedure in Appendix 7.

#### Validation of the PAOLI framework

The final draft of the framework was examined in collaboration with AK and JRS. Specifically, JRS made edits to some of the statements for clarity with PWA. The draft framework was then sent to AK who reviewed it and agreed on the final version. AK proposed the substitution of the word ‘layperson’ with ‘non-professional persons’ in the statements’ description. See Appendix 8 for examples of the edited statements.

The final draft of the PAOLI framework.

##### PAOLI phase 1: foundation

The *Foundation* involves items and statements related to the creation of a support system, for PWA, to function independently within the research team. Such items include the onset of collaborations for patient-partner recruitment, the groundwork for the induction of PWA to the project, and the delivery of adapted PPI research training sessions accustomed to the specific communication needs of PWA. The *Foundation* phase illustrates the importance of establishing functional communication by proposing the use of various communication means and the liaison with communication partners to prompt positive encounters and collaborations with the other members of the research team and to function autonomously and equally. The *Foundation* is the most extensive phase of the PAOLI framework with particular attention to the groundwork that needs to be considered by the researchers prior to the initiation of their study to involve PWA as equal partners in the research process.

##### PAOLI phase 2: development

Under the *Development* phase, items and statements highlight the importance of the meaningful involvement of PWA in the conceptualization and the identification of the topics most important to them using codesign methodology. Themes and topics should be mutually important to patient partners and the researchers to maintain shared motivation. This practice will enhance the energetic engagement of the patient partners during the co-design and co-production [37]. This will eventually improve their opportunity to participate meaningfully in the study.

##### PAOLI phase 3: translational

The content of this phase is classified based on the implementation, dissemination, and sustainability of the findings. This includes the creation of case studies for PWA to report their experiences during their involvement in the study and to suggest potential improvements in the participatory project [6]. During the *Translational* phase the dissemination of the research findings is a key topic by adding the importance of the contribution of national aphasia associations and communication support groups [30]. This phase promotes the active involvement of patient partners in disseminating research results to local aphasia organizations and international bodies representing PWA. The aim of broad dissemination is for the findings to have translational value for services related to aphasia rehabilitation.

##### PAOLI phase 4: ongoing processes

This ongoing phase includes items and statements on the provision of constant support of PWA to self-evaluate their involvement in each stage of the study [6]. This involves providing feedback on their personal experiences within the team [6] along with the monitoring of the co-design procedures. By using the PAOLI framework researchers will ensure equal opportunities for informed decision-making and guarantee the autonomous commitment of PWA within the research team [15]. Table 4 describes the final PAOLI framework with phases, items, statements, and pictograms. AK and JRS selected the pictograms from the Mulberry symbols (https://mulberrysymbols.org/) opposed to black and white line drawing infographics from the noun project website (https://thenounproject.com/).

**Table 4:** Preferred items and statements for the PAOLI framework

Phase and items		Statements description (all in close collaboration with PWA)
*Phase 1: Foundation*		
Establishing collaborations		• Make contact with local and national aphasia organisations • Establish collaborations with practice-based research networks and community clinicians
Recruitment		• Prepare a short video for patient partners’ recruitment • Develop an information leaflet in accessible format • Create an invitation letter in accessible format • Explain terminology in a way that is relevant and culturally appropriate to PWA
Gaining Informed consent		• Prepare consent form in accessible format • Enable PWA’s active involvement in informed consent procedures
Induction		• Establish rapport with PWA before entering the research group by having one-to-one introductory meetings • Explain to PWA and non-professional persons how they will be financially compensated throughout the process • Set an accessible agenda with well-defined tasks • Define how to monitor tiredness/fatigue and health status • Organise short sessions and give time for breaks • Establish the ground rules of the research team • Give PWA and non- professional persons time to familiarize themselves with the procedures
Patient Partner Training		• Prepare and present training sessions about aphasia in accessible format • Prepare and present training sessions about the research process in accessible format • Personalize training to the needs of PWA • Give PWA and non-professional persons the opportunity to ask questions for clarifications
Creating Communication Links		• Adapt communication networks and materials for culturally and linguistically diverse populations • Suggest various means of communication to be used: gestures/ signing, pictures/infographics, communication books, simplified text with bold letters, Augmentative and Alternative Communication, speech recognition software
Engaging Communication Partners		• Explain to communication partners their roles and responsibilities • Appoint a contact person for PWA
*Phase 2: Development*		
Conceptualisation		• Introduce the research team members (PWA, non-professional persons: careers, patients’ advocate) • Generate ideas from conversations and in- depth interviews • Run focus groups with PWA as facilitators to identify topics • Use different methodological approaches: learning events, personal stories groups, patients’ narratives • State the activities to be undertaken by PWA in each step, e.g., brainstorming ideas, identifying the research areas, designing the research, co- produce material, dissemination, peer interviewing and recruitment
Establishing Research Priorities		• Search for “real world” topics and PWA’s “lived‐experience” perspective • Promote patient centeredness and a focus on specific concerns • Examine the areas of concern as revealed by PWA
Reaching Consensus		• Identify the topics most important to PWA • Explore research topics of mutual interest to both scientists and PWA to strengthen research impact • Set research priorities in consensus with PWA and other non-professional persons in accessible formats • Have PWA review proposed themes • Set the research question(s) with PWA in a manner comprehensible to all partners • State why answers to these questions are important in relation to PWA’s views and opinions • Decide the research topics of mutual interest to both scientists and PWA • Make the purpose of the study easy to understand for all partners
Co-design Methodology		• Confirm that PWA and other non-professional persons assist in conducting interviews, focus groups and other selected methodologies • Define how PWA will be actively involved in co- design and co-production tasks • Define roles, responsibilities, and expectations of PWA and other non-professional persons
Proposal Development		• Clarify how PWA and non- professional persons will be actively involved in this stage • State in proposal how PWA will assist in participant recruitment • Comply with standards for ethically responsible research • Prepare documents and support material (lay summary) in collaboration with PWA and non-professional persons • List PWA as named research partners • Report the co-design and co-production methods used in collaboration with PWA and non-professional persons • Assess and state the impact of PWA and other non- professional persons involvement in the study
*Phase 3: Translational*		
Outcomes and Implementation		• Prepare dissemination videos of research outcomes • Discuss how PWA contributed to new knowledge • Discuss the outcomes of the co-learning and co-design experience • Present case studies of PWA experiences to suggest potential areas of improvements in research methodology • Discuss how the study adds to the theoretical framework of patient and public involvement in aphasia research • Implement research findings in new services related to aphasia care with the assistance of PWA • Suggest future research directions of patient and public involvement in aphasia research • State the strengths and weaknesses of such inclusive research
Disseminaton and Sustainability		• Acknowledge PWA and non-professional persons as co-authors on research publications accordingly • Acknowledge the contribution of each patient partner • Enable researchers and PWA to co-present research outcomes at scientific conferences • Disseminate outcomes in accessible formats for patient associations, newsletters, community groups, rehabilitation centers and hospitals • Involve national aphasia associations and support groups in the dissemination of results
*Phase 4: Ongoing processes*		
Support and Self-evaluation		• Support PWA to self-evaluate their engagement and personal experience
Monitoring		• Provide research updates in an accessible format for newsletters, social media posts, videos, websites etc
Impact		• State whether the involvement of PWA had an impact on their everyday life • Report the positive or negative impact of involving PWA in the research team

*The Pictograms used were downloaded freely from https://mulberrysymbols.org/ January 10th, 2022

### Dialogue model phase 5. Programming

*Programming,* an ongoing phase that was instigated from Phase 1, aims to sustain the engagement of the stakeholders. This included short informative meetings with the invited stakeholders on the purpose and commitments of the PAOLI project, online interviews with patient partners, aphasia and stroke advocates and policy makers, continuous updates of all panellists via email and with controlled feedback reports [19]. Also, during this ongoing phase*,* MC drafted the manuscript with AK and JRS as co-authors and presented the preliminary data of the study at the European Life After Stroke Forum of the Stroke Alliance for Europe (March 2023).

## Discussion

In this study the authors developed, with international consensus, the PAOLI framework, with the active involvement of PWA as PPI partners at all stages of the project. The PAOLI must be considered as a practical, PPI conceptual framework to be implemented in aphasia participatory studies. The PAOLI framework motivates to empower both researchers and PWA to build interactive, democratic, and “balanced” research teams. PAOLI includes 4 main phases, 17 items and 66 descriptive statements on how to, step by step, report on the ways of involving patient partners with aphasia in the research project. It encourages the active and meaningful involvement of PWA via codesign approaches, by providing constant support for self-reflection, and assessing the impact of their contribution to the project’s outcome and the aphasia community at large. These items were considered by the PAOLI consortium as the most important for the involvement of PWA as research partners.

### Mission statement

**Fig. 2:**
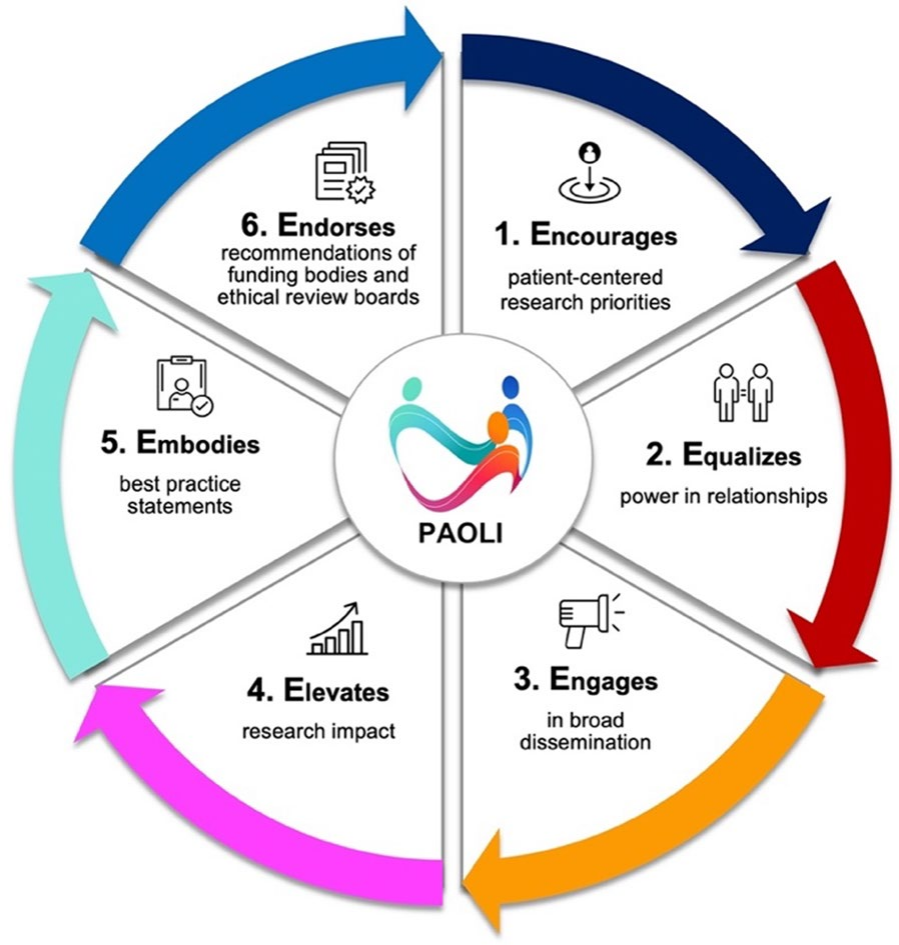
The 6 Es of the PAOLI mission statement

The PAOLI framework represents an exciting new frontier for the creation of novel PPI studies to identify aphasia rehabilitation needs and outcomes in both clinical and community settings. Placing the patient at the center of healthcare decisions has the potential to transform traditional approaches to both aphasia care and research. However, the collection and interpretation of patient generated data are not without challenges; the aphasia community will be required to invest in the work needed to ensure that the integration of the data collected following the PAOLI framework, lives up to its promise of improving the lives of PWA. PAOLI aims to empower both researchers and PWA to engage with mutual respect, and experience positive research relationships [15]. The PAOLI mission statement is characterized by six key E-verbs reported in Fig. 2 and described below.

According to the 6Es mission statement, implementing the PAOLI framework in aphasia PPI studies is crucial because it:

1. Encourages patient-centered priorities: By involving PWA as partners, research teams can ensure that the research questions, outcomes, and interventions are aligned with the needs and priorities of PWA. This patient-centered approach improves the relevance and applicability of research findings, leading to better health outcomes for patients.
2. Equalizes the power in relationships: PWA can provide valuable input during the codesign and implementation of the research. This prompts researchers to respond to the individual needs of the patient partners and share common goals.
3. Engages in broad dissemination: Patient partners with aphasia can assist in disseminating the results of the study by sharing the outcomes with their communities. Additionally, the insights and experiences of PWA within the team can enhance retention efforts by identifying strategies to improve patient partner engagement and reduce attrition rates.
4. Elevates research impact: PWA can contribute to the development of the research protocol, to tailored materials, ensuring that they are accessible and inclusive to people with communication difficulties. PWA can also help identify potential barriers or ethical considerations that researchers might overlook, leading to more robust and ethical research practices.
5. Embodies best practice statements: PWA as partners can play a crucial role in translating research findings into practice. They can help researchers communicate study results in an accessible manner, ensuring that the information is understandable, relevant, and actioned. The involvement of PWA in knowledge translation can bridge the gap between research and implementation, facilitating the uptake of evidence-based practices in clinical settings and empowering PWA to make informed decisions about their healthcare.
6. Endorses recommendations of funding bodies and ethical review boards: Including PWA in research teams promotes ethical research practices and shared decision-making. PWA can contribute to the development of research ethics guidelines, advocate for patient rights and welfare, and ensure that research aligns with patients' values and preferences. Their involvement will help to promote a more equitable and collaborative research environment and endorse research funder demands.

### PAOLI implementation to aphasia qualitative research

The PAOLI framework supports that the involvement of aphasia PPI contributors be authentic, address local needs and aim to improve overall population health and wellbeing [8]. The advantages of using the PAOLI framework while developing PROMs include the ability to capture multiple domains of importance to PWA and their carers, to increase the efficiency and reliability of data collection, and to improve sensitivity to change (responsiveness). Research and implementation priorities related to the use of the PAOLI framework while developing novel PROMs include: (1) The impact of aphasia communication impairments and other causes of patient nonresponse on the validity of PROM data; this includes research on mechanisms and techniques to improve representativeness and reduce selection bias from missing data. (2) The feasibility, use, meaning, and utility of data collected by PWA in unselected, broadly representative aphasia populations. Nyanumba and colleagues [52] stated that the cultural adaptation of self-reported tools is of great importance to ensure that involvement in research is based on situated real-life experiences in individual countries and contexts. Linking this back to the findings of our scoping review, which highlighted that very few published QoL tools involved PWA during their development, we stress the need for creating novel and culturally adaptable PROMs for PWA while implementing the PAOLI framework. (3) Finally, the constant support of PWA for self-evaluation of their contribution along with monitoring of the co-production process, will positively influence the impact of the study for both PWA and the end-product of the research.

### Specificities of the PAOLI framework in comparison to the GRIPP2

The PAOLI and the GRIPP2 aim to promote and advance the quality, transparency, and consistency of the international PPI evidence base, and to ensure best PPI practice. Jointly, GRIPP2 and the PAOLI involved PPI partners from the beginning during the conceptualization, recruitment, content selection, consensus meeting participation, validation, and finalization. Nevertheless, the GRIPP2 aims for researchers to design general PPI studies and use it as a quality assurance measure in the documentation of patient involvement while writing the scientific publication. GRIPP2 includes 34 generic items like “Describe the methods used by which patients and the public were involved” “Report the aim of the study” “Report on how PPI is used at different stages of the study” etc. In contrast, the PAOLI framework, has 17 items as an end-user targeted recommendation, aiming to help researchers support the active involvement of patients with acquired communication challenges in the research team. The main results of our study are the large number of statements (66) which were collected by our PPI approach during the PAOLI co-development phase e.g., “Identify the topics most important to PWA” and “Make the purpose of the study understandable for all partners”. These statements were codesigned to be tailored to the meaningful involvement of people with aphasia and are considered to assist both researchers and research partners in navigating a truly participatory project successfully.

The PAOLI, serves as an end-user targeted framework, aiming to help researchers support the active involvement of patients with communication challenges and acquired disabilities in the research team. PAOLI also endorses the constant support of PWA for self-evaluation. The consortium deems necessary that researchers receive constant feedback from the patient partners to avoid dropouts, inactivity, and tokenism. Additionally, although the GRIPP2 provides key items on PPI data analysis and the economic assessment of PPI; the PAOLI does not include any of these items, as they were considered by the consortium as irrelevant to the aim of this framework and the needs of the targeted population. Finally, PAOLI directs the guidance of end-users on how to generate research topics that are mutually inclusive of PWA; an area which was previously acknowledged as vague in studies including PWA.

### Scope and illustration of the PAOLI

PAOLI represents the first international evidence-based, consensus informed framework for designing and implementing PPI in aphasia research. It encompasses the issues and complexities of involving patients with persistent communication challenges and/or other disabilities within research teams. The PAOLI framework provides key PPI concepts for aphasia research that authors of future papers should incorporate, to enhance the transparency of the evidence of patient involvement. Aphasia researchers can use the PAOLI framework in advance to plan and support patient and other layperson involvement in research studies. The authors propose that researchers use the PAOLI framework to measure the impact of PPI at different stages of the research process. PAOLI provides practical guidance and actions how to transition from intention to operationalizing meaningful involvement and aims to promote aphasia advocacy and dissemination via stroke/aphasia networks. It is recommended that research studies are carried out with or by PWA through their involvement from the beginning of the research process, and in as many stages as possible. The PAOLI framework recognizes the importance of engaging those who use the healthcare system and are affected by aphasia in research to ensure that their perspectives, experiences, and needs are considered and prioritized during and after their rehabilitation. Patient and public involvement in studies on aphasia play a crucial role in shaping healthcare systems and policies, and the PAOLI framework aims to promote more relevant and responsive research through the active involvement of members with aphasia in research teams.

### PPI evidence during PAOLI development

This Delphi survey for the development of the PAOLI framework included the consistent and active involvement of two partners with chronic aphasia (AK and JRS) throughout the project timeline. JRS was involved with the recruitment of additional people with aphasia and stroke whereas AK was involved in the preparation of invitation letters, the consent forms, and the design of the of the questionnaire in accessible format. Also, AK was involved in collating the evidence and identifying topics and statements for the PAOLI framework. People with aphasia advised on the comprehensiveness, comprehensibility, and relevance of the included statements during the cognitive interviews. The communication partners (SLT students and family members) assisted PWA with the procedure for informed consent, the technical aspects of completing the cognitive interviews, and with the online Delphi questionnaires for improving the response rate in each Delphi round. Co-authors AK and JRS and a patient partner with stroke, took part in the experts meeting. Also, JRS and AK completed the validation stage of the PAOLI framework and contributed to the lay section of this paper, during the write-up phase.

The implementation of the Dialogue Model throughout the study promoted equal partnership between stakeholders and enabled patient partners to have an independent role in decision‐making for the co-development process of the PAOLI framework [25]. Nevertheless, it was not possible to demonstrate how the involvement of PWA impacted the endpoint outcome. Also, there is no published research that has demonstrated that the PPI contributions resulted in better or different outcomes [53]. We can only endorse supporting the involvement of PWA in our research teams which prompted more relevant content and resulted in a more pragmatic PPI framework. Our initial hypothesis, that the ‘lived experience’ of PWA in research teams will foster the setting of tailored research priorities and improve the content validity, can be tested in a future study. This will include researchers to codevelop a novel PROM with people with aphasia, while implementing the PAOLI framework, and compare the content with published gold standard PROM tools. For this study, the GRIPP2 checklist [20] was followed to report on patient and public involvement (see GRIPP2 checklist in Appendix 9).

### Limitations

A limitation of this study is that co-authors PPI partners did not have extensive experience with PPI projects; AK was involved in the thematic analysis [23] and in the Greek adaptation of the Aphasia Impact Questionnaire [31]. Also, the patient partners presented with diverse levels of previous exposure to research as they were recruited if they had a university degree and research experience. But this can be reported as advantageous as patient partners had dissimilar experiences in research engagement before participating in this study. Some had minor experience in research, for example the completion of a bachelor’s thesis, compared to others who had completed doctoral studies, were tenured Academics and had a vast experience in research processes and management. Also, for this study patient partners presented with mild to moderate chronic aphasia and were competent to participate (independently or with minor assistance) using written (Delphi survey) and oral communication (during the semi-structured interviews and the expert’s meeting). In addition, compared to other studies that have included PWA as research partners [6, 54, 55] we acknowledge that the 5 PWA (+ 4 patient partners with stroke and no aphasia) is a small number but not very different from previous research on similar topics as reported above. At the time, we considered the number of people with aphasia involved in this research as sufficient for the scope of this study. It’s important to stress that the PPI approach is not only new for researchers but also for PWA and this approach necessitates further understanding of research involvement practices across the research process. It is possible that the lack of financial support may have discouraged some PWA to be involved, since the project had a demanding timeline of engagement. This issue must be considered in future research.

A further limitation was the lack of funding to proceed with a more extensive, and probable face-to-face, international expert’s consensus meeting. Further obstacles throughout the process were initially a difficulty to identify global guidelines for determining consensus, sample size, and sampling techniques for the Delphi survey. The format of the “aphasia-friendly” resources in this study does not align with the published guidelines [41, 42]. Nevertheless, current research provides limited evidence of a positive effect of format modification on people with aphasia’s reading comprehension of written information [56]. Further, PWA and stroke, found it challenging to keep up with time commitments. MC noted that to send out feedback reports to panelists with communication difficulties required additional skills in written communication for messages to be ‘accessible’ to PWA. Finally, the research team experienced some challenges in developing the initial questionnaire to start the process, as there were numerous frameworks, checklists, and published resources to reference.

### Dialogue model phase 6. Implementation/future directions

For the completion of the *Implementation* phase [26] it is recommended that the PAOLI framework is tested in a future PPI aphasia study. One possibility is to implement the PAOLI framework in studies that involve people with aphasia as a primary or secondary symptom to a neurogenic/ neurodegenerative disease (e.g., dementia, parkinsonism, brain tumour/surgery, brain injuries etc.). It is recommended that the PAOLI framework is also reviewed and approved by an external board or authority [45]. Also, the authors urge stakeholders to disseminate the PAOLI framework to member meetings, networks, and scientific conferences. The authors aim to create a shorter version of the PAOLI framework to be used in studies where PPI in aphasia is a secondary focus.

## Conclusion

The PAOLI framework represents the first international evidence-based, consensus informed guide, for designing and implementing patient and public (meaningful) involvement in aphasia research. It encompasses the issues and complexities of involving patients with persistent communication impairments within research teams. The PAOLI framework aspires to improve the transparency and consistency of the international PPI aphasia inclusive research. To facilitate more effective synthesis of PPI teams, the aphasia researchers need to set up a robust support system, develop patient-relevant research questions and codesign methods adjusted to the unique needs of people with aphasia. The PAOLI framework will contribute to the advancement of understanding, knowledge, and action around meaningful involvement for exploring the impact of PPI on future aphasia research.

## Data Availability

The data generated during the current study and support the conclusions of this article are publicly available in the Appendix. Any further data queries and requests should be submitted to the corresponding author, Marina Charalambous PhD Researcher, for consideration.

## Appendices

### Appendix 2: The initial 18 items of the pilot draft with the 100 statements

PHASE 1) The Foundation

1. Establishing collaborations

How important is it:

1. To make contacts with local and national stroke and aphasia organizations.
2. To establish collaborations with practice-based research networks and community clinicians.

2. Recruitment

How important is it:

3. To prepare a short informative video for patient partners recruitment.
4. To select PWA that will contribute to the study because of a personal motive.
5. To prepare an aphasia friendly information leaflet in collaboration with PWA.
6. To prepare aphasia friendly invitation letters in collaboration with PWA.
7. To explain terminology in a way that is relevant and culturally appropriate to PWA.

3. Gaining informed consent

How important is it:

8. To prepare aphasia friendly consent forms in collaboration with PWA.
9. That PWA are actively involved in informed consent procedures using a close person that they designate.

4. Induction

How important is it:

10. To establish good rapport with PWA before entering the research group by having one-to-one introduction meetings.
11. To set a Pictorial Agenda with well-defined tasks.
12. To explain to PWA and other laypeople how they will be financially supported throughout the process.
13. To eliminate the ‘burden’ of being involved as patient partner in time consuming studies.
14. To define how are you going to monitor tiredness and fatigue/health status.
15. To organize short sessions and give time for short breaks.
16. To establish the ground rules of the research team.

5. Patient partners training

How important is it:

17. To prepare and present aphasia friendly training sessions about stroke and aphasia.
18. To prepare and present aphasia friendly training sessions about the research design and process.
19. To prepare webinars and online workshops for all members of the team.
20. To confirm that training is personalized to the needs of PWA.
21. To give opportunities to PWA and other laypeople to ask clarification questions.
22. To give opportunities to PWA and other laypeople to familiarize themselves with the procedures.

6. Creating communication links

How important is it:

23. To use online platforms and applications for direct communication with PWA.
24. To adapt *communication net*works, materials and information for culturally and linguistically diverse populations.
25. To use professional trained interpreters to promote constant engagement of PWA in the study.
26. To appoint a contact person for PWA.

7. Engaging communication partners

How important is it:

27. That PWA designate their communication partners.
28. To recruit communication partners via electronic invitation and interview them online.
29. To explain to communication partners their responsibilities.
30. To recruit healthcare students and trained volunteers when PWA cannot designate their communication partner.
31. That PWA designate as a communication partner another person with aphasia.
32. To proceed with communication skills training of communication partners.
33. To suggest various means of communication to be used e.g., total communication techniques, pictures/infographics, communication books, simplified text with bold letters, AAC devices, speech recognition software.
34. To use different methodological approaches: learning events, personal stories groups, patients’ narratives.

PHASE 2) The Development

8. Conceptualization

How important is it:

35. To introduce the research team members (PWA, carers, patients advocate, policy makers).
36. To state the activities to be undertaken by PWA in each step e.g., brainstorming ideas, identifying the research areas, designing the research, co- produce material, dissemination, peer interviewing and recruitment.
37. To generate ideas from conversations and in-depth semi-structure interviews (face-to-face or online meetings).
38. To run focus groups with PWA as facilitators to identify research topics.

9. Establishing research priorities.

How important is it:

39. To monitor dynamics in the group by giving specific responsibilities
40. To promote patient centeredness and a focus on specific concerns.
41. To search for “real‐world” and “lived‐experience perspectives”.
42. To conduct studies on common area of concern revealed by PWA.
43. To explore a research topic of mutual interest to both scientists and PWA to strengthen the impact of the research.

10. Reaching consensus

How important is it:

44. To identify topics important to PWA.
45. That PWA review proposed *themes*.
46. To set research *priorities* in consensus with PWA and other lay people.
47. To set the study title in collaboration with PWA.
48. To state in the title that it incorporates ‘patient and public involvement’ methodology.
49. That PWA present the aim of the study in an aphasia friendly manner.
50. To make sure the purpose of the study is understood by all partners.
51. To set the research question (s) with PWA in a simplified manner.
52. To state why these questions are important to be answered in relation to PWA’s *views and options*.

11. Co-design methodology

How important is it:

53. To decide on the methodology to be followed in collaboration with PWA.
54. That PWA and other laypeople assist in conducting interviews, focus groups or other selected methodology.
55. To decide on the design of the study in collaboration with PWA.
56. To define roles and expectations of each research team member.
57. To define how PWA will be involved in co-design tasks.
58. To define how the tools will be scored by PWA e.g., Patient Reported Outcome Measures- PROMS.

12. Proposal development

How important is it:

59. To ensure civically responsible and moral research.
60. To clarify how PWA and other laypeople would be actively involved in each stage.
61. To prepare documents and support material (lay summary) in aphasia friendly format.
62. That PWA be named research partners.
63. To define the involvement of PWA and other laypeople by using the PAOLI acronym and PPI throughout the text.
64. To report the co-production methods used in collaboration with PWA and other laypeople.
65. To assess and state the impact of PWA and other laypeople involvement in the research.
66. To report how PWA and other laypeople were involved during questionnaire/tool development and testing: validity, reliability, feasibility, acceptability etc.
67. PWA to assist in further participant recruitment via their network.

13. Analysis and interpretation

How important is it:

68. That PWA and other laypeople to assist in developing themes from collected data.
69. That PWA present the data to the rest of the research team.

PHASE *3) The Translational*

14. Outcomes and implementation

How important is it:

70. To prepare dissemination videos of research outcomes in collaboration with PWA.
71. *To discuss how PWA contributed to new knowledge*.
72. To discuss the outcomes of the co-learning and co-producing experience.
73. To present case studies of PWA experiences to indicated potential benefits in terms of improvements in research.
74. To discuss how the study adds to the theoretical framework of PPI in aphasia research.
75. To implement research findings in new services related to Aphasia assisted by PWA.
76. To state the strengths and weaknesses of such an inclusive research model.
77. To discuss of the economic cost or benefit of such an inclusive model.
78. To suggest future research directions of PPI in aphasia research.

15. Dissemination and sustainability

How important is it:

79. That PWA prepare dissemination events via their informal networks
80. That researchers and PWA present the research outcomes to scientific conferences.
81. To disseminate outcomes in aphasia friendly format for local patient associations, newsletters, community groups, rehabilitation centers and hospitals.
82. To involve national aphasia associations and stroke support organizations for dissemination of the results.
83. *To* acknowledge the contribution of each patient research partner.
84. To acknowledge by name PWA and other layperson as co-authors on research publication.

PHASE 4: Ongoing Processes

16. Support, reflection and self-evaluation

How important is it:

85. To involve regular meetings of reflection *groups*.
86. To offer peer support, mentoring, coaching or virtual support sessions.
87. That PWA and other laypeople prepare a reflection logbook and address issues as they arise.
88. That PWA can self-evaluate their involvement and personal experience in the study

17. Monitoring

How important is it:

89. That PWA form a committee to check the progress of the research.
90. To review all consent forms and information material with PWA.
91. To provide research updates in an aphasia friendly format for newsletters, social media posts, videos, leaflets

18. Impact

How important is it:

92. To report the positive or negative impact of involving PWA in the research team.
93. To report the wider social impact of the involvement of PWA in the study.
94. To report the economic impact of the involvement of PWA in the study.
95. To state how the involvement of PWA had an impact on their quality of life.
96. To discuss how contextual factors influenced the involvement of PWA in the study (constraints imposed by organisations or funders may lead to tokenistic involvement and reduced impacts).
97. To discuss how environmental factors influenced the involvement of PWA in the study (well- supported involvement is more likely to have desired impacts).
98. To discuss how personal factors influenced the involvement of PWA in the study (personal motivation, confidence, increased skills and knowledge).
99. To discuss how the involvement of PWA in the study influenced their activity and participation level.
100. To state clearly how PWA and other laypeople involvement meets funder demands.

### Appendix 4: Recommendations for the Conducting and REporting of DElphi Studies (CREDES) Checklist

**Table:**

Items of reporting	Reported on page
*Purpose and rationale.* The purpose of the study should be clearly defined and demonstrate the appropriateness of the use of the Delphi technique as a method to achieve the research aim. A rationale for the choice of the Delphi technique as the most suitable method needs to be provided	9,16
*Expert panel.* Criteria for the selection of experts and transparent information on recruitment of the expert panel, sociodemographic details including information on expertise regarding the topic in question, (non)response and response rates over the ongoing iterations should be reported	18–20
*Description of the methods.* The methods employed need to be comprehensible; this includes information on preparatory steps (How was available evidence on the topic in question synthesized?), piloting of material and survey instruments, design of the survey instrument(s), the number and design of survey rounds, methods of data analysis, processing and synthesis of experts’ responses to inform the subsequent survey round and methodological decisions taken by the research team throughout the process	16–19
*Procedure.* Flow chart to illustrate the stages of the Delphi process, including a preparatory phase, the actual ‘Delphi rounds’, interim steps of data processing and analysis, and concluding steps	Appendix 7
*Definition and attainment of consensus.* It needs to be comprehensible to the reader how consensus was achieved throughout the process, including strategies to deal with non-consensus	20–21
*Results*. Reporting of results for each round separately is highly advisable in order to make the evolving of consensus over the rounds transparent. This includes figures showing the average group response, changes between rounds, as well as any modifications of the survey instrument such as deletion, addition or modification of survey items based on previous rounds	21–30
*Discussion of limitations.* Reporting should include a critical reflection of potential limitations and their impact of the resulting guidance	38,39
*Adequacy of conclusions.* The conclusions should adequately reflect the outcomes of the Delphi study with a view to the scope and applicability of the resulting practice guidance	40
*Publication and dissemination*. The resulting guidance on good practice in palliative care should be clearly identifiable from the publication, including recommendations for transfer into practice and implementation. If the publication does not allow for a detailed presentation of either the resulting practice guidance or the methodological features of the applied Delphi technique, or both, reference to a more detailed presentation elsewhere should be made (e.g., availability of the full guideline from the authors or online; publication of a separate paper reporting on methodological details and particularities of the process (e.g. persistent disagreement and controversy on certain issues). A dissemination plan should include endorsement of the guidance by professional associations and health care authorities to facilitate implementation	39,40

### Appendix 5: The median scores of the statements voted with a positive consensus in each round

**Table:**

Phase and items	Statement description	Round one scores			Round two scores			Consensus
		Median	Mode	% of total	Median	Mode	% of total	
*Phase 1. The foundation*								
1. Establishing collaborations	Make contact with local and national aphasia organisations	5.00	5.00	95	5.00	5.00	95	**√**
	Establish collaborations with practice-based networks and community clinicians	5.00	5.00	90	5.00	5.00	90	**√**
2. Recruitment	Prepare a short informative video for patient recruitment	4.00	4.00	80	5.00	5.00	90	**√**
	Develop an aphasia friendly information leaflet in collaboration with PWA	5.00	5.00	95	5.00	5.00	95	**√**
	Create aphasia friendly invitation letters in collaboration with PWA	5.00	5.00	90	5.00	5.00	90	**√**
	Explain terminology in a way that is relevant and culturally appropriate to PWA	5.00	5.00	80	5.00	5.00	90	**√**
3. Gaining informed consent	Prepare aphasia friendly consent forms in collaboration with PWA	5.00	5.00	90	5.00	5.00	80	**√**
	Ensure that PWA are actively involved in informed consent procedures using a close person that they designate	4.00	4.00	80	4.00	4.00	80	**√**
4. Induction	Establish rapport with PWA before entering the research group by having one-to-one introductory meetings	5.00	5.00	85	4.00	4.00	80	**√**
	Explain to PWA and non-professional persons how they will be financially compensated throughout the process	4.00	5.00	80	4.00	5.00	80	**√**
	Set an accessible agenda with well-defined tasks in collaboration with PWA	4.00	4.00	80	4.00	4.00	85	**√**
	Define how to monitor tiredness/fatigue and health status in collaboration with PWA	4.50	5.00	80	5.00	5.00	80	**√**
	Organise short sessions and give time for breaks	4.50	5.00	80	4.50	5.00	85	**√**
	Establish the ground rules of the research team in collaboration with PWA	5.00	5.00	80	5.00	5.00	90	**√**
5. Patient partner training	Prepare and present aphasia friendly training sessions about the research design and process	5.00	5.00	90	5.00	5.00	95	**√**
	Prepare and present aphasia friendly training sessions about the stroke and aphasia	5.00	5.00	85	5.00	5.00	85	**√**
	Confirm that the training is accessible to the communication needs of PWA	4.00	5.00	90	4.00	5.00	95	**√**
	Give PWA and non-professional persons the opportunity to ask questions for clarification	5.00	5.00	85	5.00	5.00	85	**√**
	Give PWA and non- professional persons time to familiarize themselves with the procedures	4.00	4.00	80	4.00	5.00	80	**√**
6. Create communication links	Adapt communication networks and materials for culturally and linguistically diverse populations	4.00	4.00	75	4.00	4.00	85	**√**
	Suggest various means of communication to be used: gestures/ signing, pictures/infogra., communication books, simplified text with bold letters, Augmentative and Alternative Communication, speech recognition software	4.00	5.00	80	4.00	5.00	80	**√**
7. Engaging communication partners	Explain to communication partners their roles and responsibilities	4.00	5.00	75	5.00	5.00	85	**√**
	Appoint a contact person for PWA	4.00	4.00	85	4.00	5.00	85	**√**
*Phase 2. The development*								
8. Conceptualization	Introduce the research team members (PWA, non-professional persons: carers, patients’ advocates)	5.00	4.00	85	5.00	5.00	90	**√**
	Generate ideas from conversations and in- depth interviews	5.00	5.00	90	5.00	5.00	90	**√**
	Run focus groups with PWA as facilitators to identify topics	4.00	4.00	80	4.00	5.00	90	**√**
	Use different methodological approaches: learning events, personal stories groups, patients’ narratives	4.00	4.00	75	5.00	4.00	85	**√**
	State the activities to be undertaken by PWA in each step, e.g., brainstorming ideas, identifying the research areas, designing the research, co- produce material, dissemination, peer interviewing and recruitment	5.00	5.00	80	5.00	5.00	80	**√**
9. Establishing research priorities	Search for “real world” topics and PWA’s “lived‐experience” perspective	4.00	5.00	80	5.00	5.00	85	**√**
	Promote patient centeredness and a focus on specific concerns	5.00	5.00	90	5.00	5.00	95	**√**
	Examine the areas of concern as revealed by PWA	5.00	4.00	90	5.00	5.00	85	**√**
10.Reaching consensus	Identify the topics most important to PWA	5.00	5.00	100	5.00	5.00	100	**√**
	Explore research topics of mutual interest to both scientists and PWA to strengthen research impact	5.00	5.00	75	5.00	5.00	85	**√**
	Set research priorities in consensus with PWA and other non-professional persons in aphasia-friendly formats	4.00	5.00	80	4.00	4.00	95	**√**
	Have PWA review proposed themes	4.00	5.00	80	4.00	5.00	85	**√**
	Set the research question(s) with PWA in a manner comprehensible to all partners	4.00	5.00	95	4.00	4.00	95	**√**
	State why answers to these questions are important in relation to PWA’s views and opinions	4.00	5.00	80	4.00	5.00	85	**√**
	Decide the research topics of mutual interest to both scientists and PWA to strengthen the impact of the research	5.00	5.00	85	5.00	5.00	85	**√**
	Ensure that the purpose of the study is understood by all partners	5.00	5.00	100	5.00	5.00	100	**√**
11. Co-design methodology	Confirm that PWA and other non-professional persons assist in conducting interviews, focus groups and other selected methodology	4.00	4.00	75	5.00	5.00	85	**√**
	Define how PWA will be actively involved in co- design tasks	4.00	4.00	70	4.00	4.00	90	**√**
	Define roles, responsibilities, and expectations of PWA and other non-professional persons	4.00	4.00	85	4.00	4.00	85	**√**
12. Proposal development	Clarify in the proposal how PWA and non- professional persons will be actively involved in this stage of the study	4.00	5.00	85	4.00	5.00	80	**√**
	State how PWA will assist in participant recruitment	4.00	5.00	75	4.00	4.00	85	**√**
	Ensure ethically responsible research	5.00	5.00	90	5.00	5.00	90	**√**
	Prepare documents and support material (lay summary) in collaboration with PWA and non-professional persons	4.00	5.00	80	4.00	5.00	85	**√**
	Ensure that PWA are named research partners	5.00	5.00	90	5.00	5.00	90	**√**
	Report the co- design and co-production methods used in collaboration with PWA	4.00	5.00	75	5.00	5.00	90	**√**
	Assess and state the impact of PWA and other non- professional persons involvement in the study	4.00	4.00	70	4.00	5.00	90	**√**
*Phase 3. The Translational*								
13. Outcomes and implementation	Prepare dissemination videos of research outcomes	4.00	5.00	85	4.00	5.00	85	**√**
	Discuss how PWA contributed to new knowledge	4.00	5.00	80	4.00	5.00	90	**√**
	Discuss the outcomes of the co-learning and co-design experience	4.00	5.00	80	4.00	5.00	80	**√**
	Present case studies of PWA experiences to suggest potential areas of improvements in research methodology	4.00	5.00	90	4.00	5.00	90	**√**
	Discuss how the study adds to the theoretical framework of patient and public involvement in stroke and aphasia research	4.00	4.00	85	4.00	5.00	85	**√**
	Implement research findings in new services related to stroke and aphasia with the assistance of PWA	4.00	4.00	80	4.00	4.00	80	**√**
	Suggest future research directions of patient and public involvement in stroke and aphasia research	4.00	4.00	80	4.00	5.00	80	**√**
	State the strengths and weaknesses of such inclusive research model	4.00	5.00	80	4.00	5.00	80	**√**
14. Dissemination and sustainability	Acknowledge PWA and non-professional persons as co-authors on research publication accordingly	4.00	4.00	80	4.00	4.00	80	**√**
	Acknowledge the contribution of each patient partner	4.00	4.00	80	4.00	5.00	80	**√**
	Enable researchers and PWA to co-present research outcomes at scientific conferences	4.00	4.00	85	4.00	4.00	90	**√**
	Disseminate outcomes in aphasia-friendly formats for patient associations, newsletters, community groups, rehabilitation centers and hospitals	5.00	5.00	85	5.00	5.00	95	**√**
	Involve national aphasia associations and stroke support groups in the dissemination of results	5.00	5.00	90	5.00	5.00	95	**√**
*Phase 4. Ongoing processes*								
15. Support and self-evaluation	Support PWA to self-evaluate their involvement and personal experience in the study	4.00	5.00	75	4.00	5.00	85	**√**
16. Monitoring	Provide research updates in an aphasia friendly format for newsletters, social media posts, videos, leaflets etc	4.00	5.00	80	5.00	5.00	85	**√**
17. Impact	State whether the involvement of PWA had an impact on their quality of life	4.00	4.00	85	4.00	4.00	85	**√**
	Report the positive or negative impact of involving PWA in the research team	4.00	5.00	85	4.00	5.00	85	√

### Appendix 6: Consensus meeting outcomes

**Table:**

Item and statement	Outcomes of discussion
Item: Data Analysis and Interpretation Statement: That PWA and other laypeople assist in analyzing the collected data	All experts agreed that this item and the proposed statement should not be be included in the PAOLI framework and that patient partners should not be involved in data analysis. Patient partners that were involved in the experts meeting confirmed that data analysis is a difficult task for people with aphasia
Item: Data Analysis and Interpretation Statement: That PWA present the data to the rest of the research team	All experts agreed this item and the proposed statement should not be included and that patient partners should not be involved in presenting data unless is stated in the purpose of the study. Patient partners that were involved in the experts meeting confirmed that presenting data is very challenging and linguistically demanding for people with aphasia
Item: Outcomes and Implementation Statement: To discuss the economic parameters of such an inclusive model	All experts agreed that the proposed statement of this item should be excluded from the PAOLI framework as it is not relevant and/or appropriate in all countries
Item: Impact Statement: To state whether PWA and other laypeople involvement met funder demands	All experts agreed this item is not included as it is not relevant for all countries, and it is complicated to assess

### Appendix 8 Examples of the statements edited by PPI partners JRS and AK during the PAOLI validation.

**Table:**

Item	Voted statement	Reviewed statement	Rationale
Induction	Explain to PWA and other laypeople how they will be financially supported throughout the process	Explain to PWA and non-professional persons how they will be financially compensated throughout the process	Replace the word ‘supported’ with ‘compensated’ as “compensated” refers to their collaboration whereas “supported” directs in the financial support of one’s total cost of living
Proposal development	Ensure civic ethics and moral research	Ensure ethically responsible research	The meaning of “civically” responsible research is not clear and whether research is moral or not, rather than ethical
Reaching consensus	Define the research topics of mutual interest to both scientists and PWA to strengthen the impact of the research	Decide the research topics of mutual interest to both scientists and PWA to strengthen the impact of the research	Replace the word ‘define’ with ‘decide’ as we have done the “exploration” in previous sections and here we need to decide what topics will be pursued

### Appendix 9 The Guidance for Reporting Involvement of Patients and Public 2 Long Form (GRIPP2-LF) reporting checklist

**Table:**

Section and topic	Item	Reported on page No
Section "Introduction": Abstract of paper		
1a: Aim	Report the aim of the study	2
1b: Methods	Describe the methods used by which patients and the public were involved	2
1c: Results	Report the impacts and outcomes of PPI in the study	2
1d:Conclusions	Summarise the main conclusions of the study	2
1e: Keywords	Include PPI, “patient and public involvement,” or alternative terms as keywords	2
Section "Methods": Background to paper		
2a: Definition	Report the definition of PPI used in the study and how it links to comparable studies	4
2b: Theoretical underpinnings	Report the theoretical rationale and any theoretical influences relating to PPI in the study	4–7
2c: Concepts and theory development	Report any conceptual or theoretical models, or influences, used in the study	4–7
Section "Results": Aims of paper		
3: Aim	Report the aim of the study	8
Section "Discussion": Methods of paper		
4a: Design	Provide a clear description of methods by which patients and the public were involved	9–10
4b: People involved	Provide a description of patients, carers, and the public involved with the PPI activity in the study	11–20
4c: Stages of involvement	Report on how PPI is used at different stages of the study	37, 38
4d: Level or nature of involvement	Report the level or nature of PPI used at various stages of the study	9–20, 37, 38
Section "Conclusion": Capture or measurement of PPI impact		
5a: Qualitative evidence of impact	If applicable, report the methods used to qualitatively explore the impact of PPI in the study	n/a
5b: Quantitative evidence of impact	If applicable, report the methods used to quantitatively measure or assess the impact of PPI	n/a
5c: Robustness of measure	If applicable, report the rigour of the method used to capture or measure the impact of PPI	n/a
Section "Creating communication links": Economic assessment		
6: Economic assessment	If applicable, report the method used for an economic assessment of PPI	n/a
Section "Engaging communication partners": Study results		
7a: Outcomes of PPI	Report the results of PPI in the study, including both positive and negative outcomes	26–28
7b: Impacts of PPI	Report the positive and negative impacts that PPI has had on the research, the individuals involved (including patients and researchers), and wider impacts	29–31
7c: Context of PPI	Report the influence of any contextual factors that enabled or hindered the process or impact of PPI	37–39
7d: Process of PPI	Report the influence of any process factors, that enabled or hindered the impact of PPI	37–39
7ei: Theory development	Report any conceptual or theoretical development in PPI that have emerged	31–34
7eii: Theory development	Report evaluation of theoretical models, if any	33–35
7f: Measurement	If applicable, report all aspects of instrument development and testing (eg. validity, reliability, feasibility, acceptability, responsiveness, interpretability, appropriateness, precision)	10–13, 22, 23
7 g: Economic assessment	Report any information on the costs or benefit of PPI	n/a
Section "Conceptualization": Discussion and conclusions		
8a: Outcomes	Comment on how PPI influenced the study overall. Describe positive and negative effects	29–31
8b: Impacts	Comment on the different impacts of PPI identified in this study and how they contribute to new knowledge	33
8c: Definition	Comment on the definition of PPI used (reported in the Background section) and whether or not you would suggest any changes	33
8d: Theoretical underpinnings	Comment on any way your study adds to the theoretical development of PPI	33–35
8e: Context	Comment on how context factors influenced PPI in the study	38, 39
8f: Process	Comment on how process factors influenced PPI in the study	38, 39
8g: Measurement and capture of PPI impact	If applicable, comment on how well PPI impact was evaluated or measured in the study	n/a
8h: Economic assessment	If applicable, discuss any aspects of the economic cost or benefit of PPI, particularly any suggestions for future economic modelling	n/a
8i: Reflections/critical perspective	Comment critically on the study, reflecting on the things that went well and those that did not, so that others can learn from this study	38, 39

## Abbreviations

PPI: Patient and Public Involvement
PWA: People With Aphasia
GRIPP: Guidance for Reporting Involvement of Patients and the Public
PAOLI: People with Aphasia and Other Layperson Involvement
MC: Marina Charalambous
AK: Alexia Kountouri
JRS: Jürg Rainer Schwyter
JMA: Jean-Marie Annoni
MK: Maria Kambanaros
EQUATOR: Enhancing the QUAlity and Transparency Of health Research
ASRS: Aphasia Severity Rating Scale
BDAE: Boston Diagnostic Aphasia Examination
SSwoA: Stroke Survivors without Aphasia

## Footnotes

1 MC completed a course on ‘Patient Involvement in Research’ at the School of Public Health at Imperial College London (in November 2021).
